# Supplementary figures and images for: Genetic and potential antigenic evolution of influenza A(H1N1)pdm09 viruses circulating in Kenya during 2009–2018 influenza seasons
Source: Sci Rep. 2023 Dec 15;13:22342. doi: 10.1038/s41598-023-49157-3 (PMC10724140; doi:10.1038/s41598-023-49157-3)

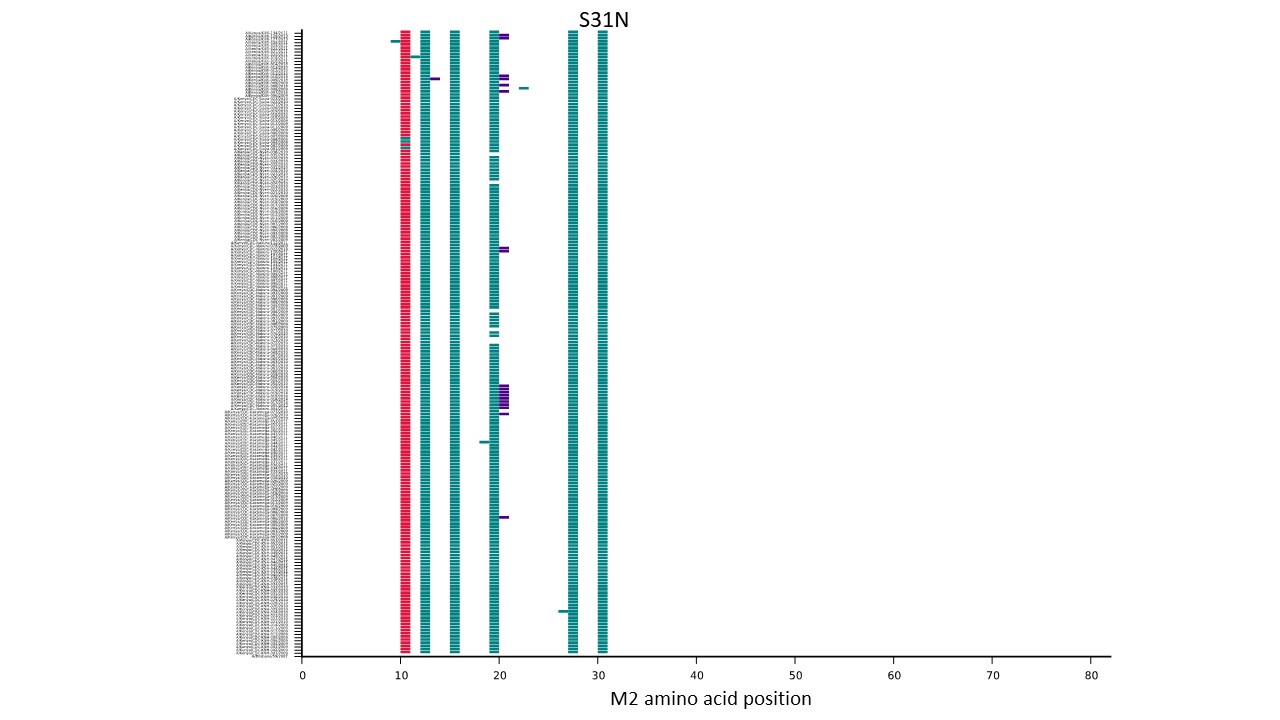

Supplement: Supplementary file 1 — Supplementary Figure S1. [file 41598_2023_49157_MOESM1_ESM.jpg]

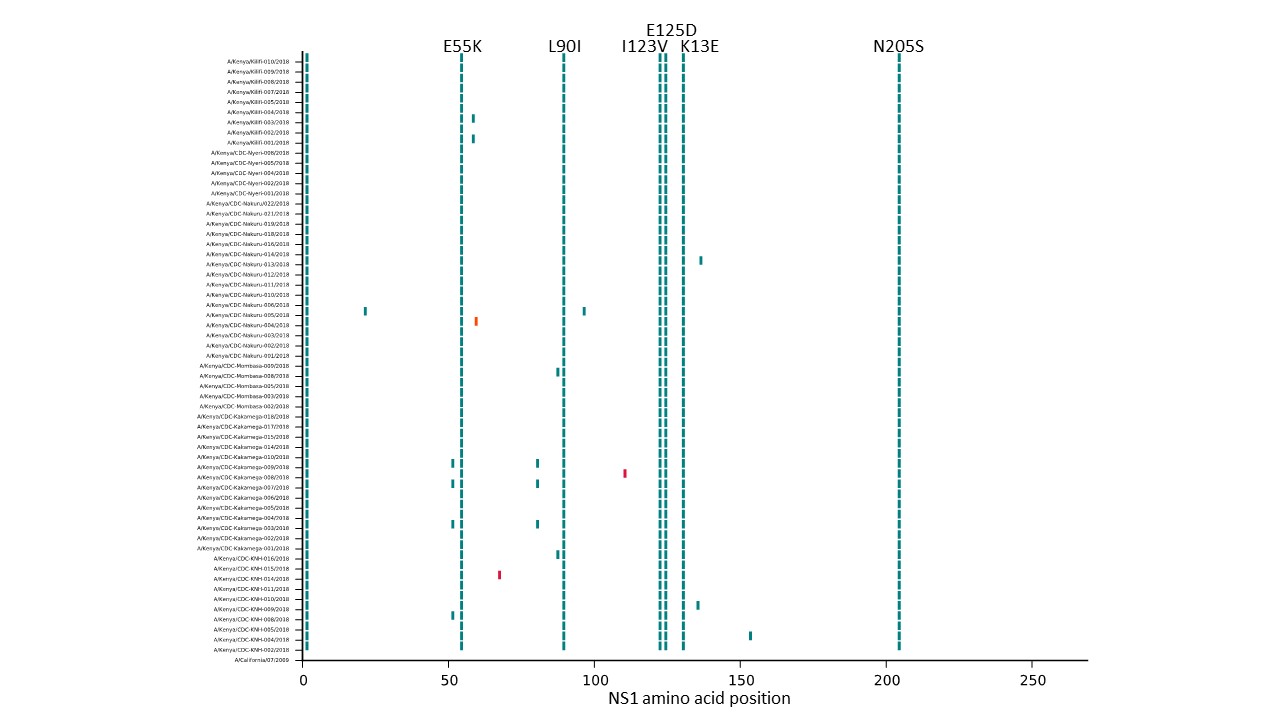

Supplement: Supplementary file 2 — Supplementary Figure S2. [file 41598_2023_49157_MOESM2_ESM.jpg]
