# Supplementary material for: Genetic and potential antigenic evolution of influenza A(H1N1)pdm09 viruses circulating in Kenya during 2009–2018 influenza seasons
Source: Sci Rep. 2023 Dec 15;13:22342. doi: 10.1038/s41598-023-49157-3 (PMC10724140; doi:10.1038/s41598-023-49157-3)
Supplement: Supplementary file 3 — Supplementary Legends. [file 41598_2023_49157_MOESM3_ESM.docx]

**Supplementary Figure Legends**

**Figure S1.** Highlighter plot of M2 protein sequences of A(H1N1)pdm09 viruses from Kenya showing the adamantine-resistance marker S31N amino acid substitution in M2 protein. The substitution was observed in all the A(H1N1)pdm09 viruses collected in Kenya between 2009 and 2018. Sequences were aligned with A/Brisbane/59/2007.

**Figure S2.** Highlighter plot of NS1 protein sequences of A(H1N1)pdm09 viruses from Kenya showing the NS1 protein substitutions: E55K, L90I, I123V, E125D, K131E, and N205S. The sequences were aligned with vaccine strain A/California/07/2009. The substitutions were reported for 59 virus sequences collected from hospitalized patients in 2018 from CDC-supported surveillance and KCH pediatric viral pneumonia surveillance. CDC, Centers for Disease Control; KCH, Kilifi County Hospital.
